# Parent-reported outcomes in young children with disorders/differences of sex development

**DOI:** 10.1186/s13633-020-0073-x

**Published:** 2020-02-14

**Authors:** Salma R. Ali, Zoe Macqueen, Melissa Gardner, Yiqiao Xin, Andreas Kyriakou, Avril Mason, M. Guftar Shaikh, Sze C. Wong, David E. Sandberg, S. Faisal Ahmed

**Affiliations:** 1 Developmental Endocrinology Research Group, School of Medicine, Dentistry & Nursing, University of Glasgow, Glasgow, UK; 2 Susan B Meister Child Health Evaluation and Research Center, Department of Pediatrics, University of Michigan Medical School, Ann Arbor, Michigan USA; 3 Health Economics & Health Technology Assessment, Institute of Health and Wellbeing, University of Glasgow, Glasgow, UK

**Keywords:** DSD, Psychosocial adjustment, PRO, Screening

## Abstract

**Background:**

There is a paucity of tools that can be used in routine clinical practice to assess the psychosocial impact of Disorders/Differences of Sex Development (DSD) on parents and children.

**Objective:**

To evaluate the use of short Parent Self-Report and Parent Proxy-Report questionnaires that can be used in the outpatient setting.

**Methods:**

Previously validated DSD-specific and generic items were combined to develop a Parent Self-Report questionnaire and a Parent Proxy-Report questionnaire for children under 7 years. Of 111 children approached at one tertiary paediatric hospital, the parents of 95 children (86%) with DSD or other Endocrine conditions completed these questionnaires.

**Results:**

Questionnaires took under 10 min to complete and were found to be easy to understand. Compared to reference, fathers of children with DSD reported less stress associated with Clinic Visits (*p* = 0.02) and managing their child’s Medication (*p* = 0.04). However, parents of children with either DSD or other Endocrine conditions reported more symptoms of Depression (*p* = 0.03). Mothers of children with DSD reported greater Future Concerns in relation to their child’s condition (median SDS − 0.28; range − 2.14, 1.73) than mothers of children with other Endocrine conditions (SDS 1.17; − 2.00, 1.73) (*p* = 0.02). Similarly, fathers of children with DSD expressed greater Future Concerns (median SDS -1.60; − 4.21, 1.00) than fathers of children with other Endocrine conditions (SDS 0.48; − 2.13, 1.52) (*p* = 0.04).

**Conclusion:**

DSD was associated with greater parental concerns over the child’s future than other Endocrine conditions. Brief parent-report tools in DSD can be routinely used in the outpatient setting to assess and monitor parent and patient needs.

## Introduction

Conditions that affect somatic sex development such as Differences/Disorders of Sex Development (DSD) can impose a high degree of stress on patients and their families and exert a wide range of effects on ‘social and psychosexual adjustment, mental health, quality of life and social participation’ [1–3]. Distress related to shame and ‘stigma’ associated with DSD, uncertainties regarding diagnosis and limited coping strategies may lead to an increased risk of adverse psychosocial outcomes disproportionate to the severity of the DSD [4–7]. A significant minority of parents of children with DSD experience symptoms suggestive of post-traumatic stress disorder with accompanying difficulties in communicating news of their child’s condition with relatives and close friends, an independent risk factor for emotional distress [8, 9]. Yet there is a paucity of studies evaluating the feasibility of routine, longitudinal screening for challenges to positive psychosocial adaptation in the context of usual care [10–12]. Such screening would ideally go beyond the heretofore restricted focus on psychosexual differentiation, i.e., gender identity, gender role, and sexual orientation [11, 13].

Generally, there has been growing interest in adopting standardised tools for assessing subjective experiences of patients and incorporating reports of parent/caregiver proxies in young children in the context of ongoing patient care [14, 15]. The assessment of a child’s adaptation to their medical condition is also becoming increasingly common [16] with the use of parent-proxy reporting playing an important role in overcoming challenges associated with assessing the subjective experience of young children [17]. Parent/patient reported outcome (PRO) measures can be generic (i.e., applicable to all populations), such as the scales comprising the ‘Patient-Reported Outcomes Measurement Information System’ (PROMIS®) [18] and the ‘Patient Health Questionnaire for Depression and Anxiety’ [19] or can be condition-specific, for example, the ‘Pediatric Asthma Scale’ [20]. A methodological gap in developing such tools was recently addressed by the development of DSD-specific health-related quality of life measures for parents of children under 7 years of age including a parent-proxy measure and a self-report measure [4]. Whereas these tools were developed for use within multidisciplinary DSD clinics with dedicated behavioural health specialists, it is unclear whether their use would be feasible in settings with more limited staffing and time constraints. There is a need to explore tools that can overcome the perceived challenges of managing patients in a busy clinic setting and have maximum acceptability by parents and professionals [21, 22].

The purpose of this project was to develop a self-report questionnaire for parents of children aged from birth to under 7 years and a parallel proxy-report questionnaire for parents of children aged 2 to < 7 years, using existing validated DSD-specific [4, 5] and generic items [18], for parents of young children, that can be routinely used in a busy outpatient setting to assess the impact of DSD on parents and their children.

## Methods

### Parents

**Fig. 1:**
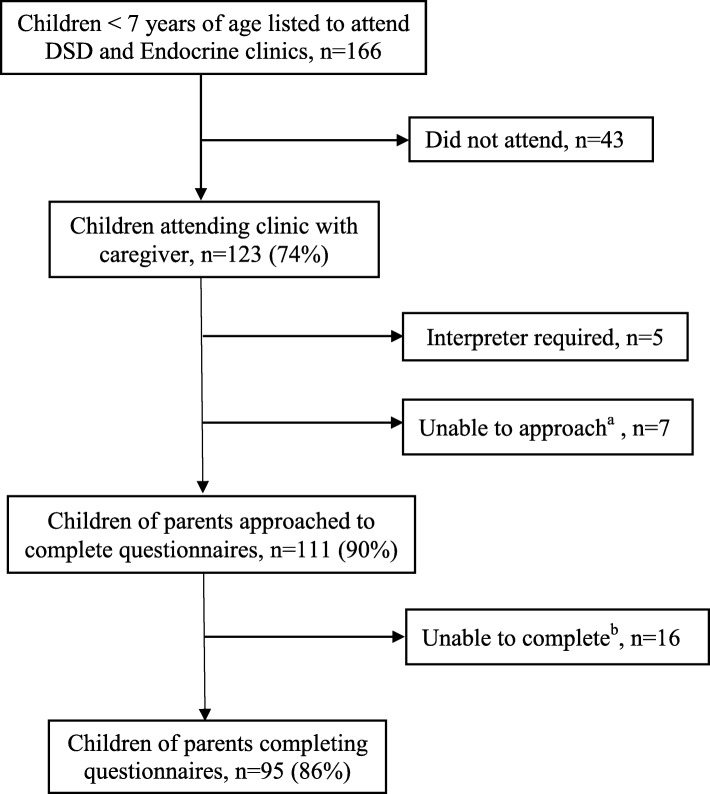
Cases and recruitment details. DSD, Disorder of Sex Development. ^a^Caregivers not approached due to consultation running simultaneously (*n* = 7) ^b^Caregivers not able to complete questionnaires as insufficient time after clinic (*n* = 8), other commitments (n = 7) or already completed a separate hospital questionnaire (n = 1)

Parents of children under the age of 7 years attending DSD and Endocrine outpatient clinics at one specialist paediatric centre, were approached between February 2017 and February 2019 (Fig. 1). Exclusion criteria included the need for an interpreter for questionnaire completion, thus, parental eligibility was restricted to those whose primary language was English. Parents were approached at the end of clinic consultations, advised verbally and provided with a cover note that their participation was optional and that the completed questionnaires would be included in their child’s health record as part of routine clinical care. Questionnaires were provided to one parent/carer attending clinic with their child with the exception of four cases for whom both mothers and fathers completed questionnaires. Only two of the respondents who completed the questionnaires were not biological parents of the children, but all were referred to as ‘mothers’ and ‘fathers’. All parents returned completed questionnaires prior to leaving the clinic. A section at the end of the questionnaire sought parental feedback on simplicity of the questionnaire, on acceptability of the length of time for completion and on comprehension of questions. The questionnaire was approved by the research ethics office as part of routine healthcare evaluation. The completed questionnaires were available to the DSD multidisciplinary team assessing care and for discussion at subsequent pre-clinic meetings for evaluation of ongoing care.

### Parent self-report (PSR) questionnaire

The 40-item PSR (Additional file 1: Table S1, Additional file 1: Figure S1) was developed for parents of children aged from birth to < 7 years to assess parental feelings and experiences in relation to their child’s condition (DSD or other Endocrine conditions). The PSR is comprised of 8 domains (Healthcare Communication and Information, Talking to Others, Future Concerns, Medication, Clinic Visit, Surgery, Stigma, and Anxiety/Depression) selected from the previously validated Quality of Life DSD Parent (QOL-DSD-Parent) report [4], parent-focused items of the Experiences and Reactions questionnaire assessing DSD-related experienced or anticipated stigma [5], and the Patient Health Questionnaire-4 (PHQ-4) [19], a screening scale for depression and anxiety (Table 1).

**Table 1:** PRO questionnaire domains, score representation and reference mean (SD)

Questionnaire Domains	Items	Domains derived from:	High subscale scores indicate:	Sample mean (SD) from reference sample^a,b^			Reference sample
				Mothers	Fathers	Sample- All	
Parent Self-Report							
Communication and Information	2	QOL-DSD	Better outcome	74.86 (16.93)	69.97 (23.15)	–	Alpern et al. (2017)^4^
Talking to Others	5	QOL-DSD	Better outcome	64.03 (24.52)	85.55 (16.80)	–	
Future Concerns	7	QOL-DSD	Better outcome	55.37 (25.86)	79.14 (13.71)	–	
Medication	4	QOL-DSD	Better outcome	70.39 (28.20)	49.10 (28.31)	–	
Clinic Visit	4	QOL-DSD	Better outcome	72.08 (27.81)	33.38 (25.80)	–	
Surgery	4	QOL-DSD	Better outcome	38.03 (25.12)	81.10 (24.51)	–	
Stigma	10	Experiences & Reactions: Parent focused	Poorer outcome	1.76 (0.63)	1.56 (0.44)	–	Rolston et al. (2015)^5^
PHQ-4	4	Patient Health Questionnaire-4	Poorer outcome	–	–	2.5 (2.8)	Kroenke et al. (2009)^19^
Parent Proxy-Report							
Anxiety	4	PROMIS	Poorer outcome	–	–	50 (10)	Irwin et al., (2012)^18^
Depression	4	PROMIS	Poorer outcome	–	–	50 (10)	
Anger	5	PROMIS	Poorer outcome	–	–	50 (10)	
Peer Relations	4	PROMIS	Better outcome	–	–	50 (10)	
Stigma	4	Experiences & Reactions: Child focused	Poorer outcome	2.28 (0.91)	2.05 (0.81)	–	Rolston et al. (2015)^5^
Clinic Visit & Medication	7 1	QOL-DSD	Better outcome	64.98 (24.49)	78.10 (22.56)	–	Alpern et al. (2017)^4^

^a^Sample mean (SD) from reference data was used to calculate median SDS for each domain [4, 5, 18, 19]. ^b^Mean (SD) represents the mean (SD) T score for PROMIS domains

### Parent proxy-report (PPR) questionnaire

The 30-item PPR (Additional file 1: Table S2, Additional file 1: Figure S2) was developed for parents of children aged 2 to < 7 years to capture their perceptions of the child’s feelings and experiences related to their condition (DSD or other Endocrine condition). Paralleling construction of the PSR, the PPR comprises 8 domains (Anxiety, Depression, Anger, Peer Relationships, Stigma, Clinic Visits, Medications, and Missed School Days) selected from the Quality of Life DSD Proxy (QOL-DSD-Proxy) report [4], child-focused items of the Experiences and Reactions questionnaire [5] and select PROMIS Parent-Proxy scales [18] (Table 1).

### Questionnaire scoring

DSD reference data were obtained from a previous study validating DSD-specific, parent-reported, health-related quality of life measures for children under the age of 7 years [4]. For both PSR and PPR questionnaires, parents rated their experiences/perceptions on 5-point Likert scales (Additional file 1: Tables S1 and S2). For each domain, a scale average was obtained if at least half of the items were completed, and this was incorporated into the calculation of the median standard deviation score (SDS). Responses were standardised from 0 to 100, with higher scores in the majority of domains indicating better quality of life and more positive adaptation (Table 1). The Experiences and Reactions (Stigma) domain [5] was also scored on 5-point scale, with higher scores indicating a greater level of experienced (or anticipated) stigma. Median SDS, i.e., the number of SDs away from the mean, for questionnaire domains stemming from the QOL-DSD-Parent, QOL-DSD-Proxy reports [4] and the Experiences and Reaction (Parent-Focused and Child-Focused) questionnaire [5], were calculated using separate mother and father means and SDs obtained from reference data (Table 1). Total scores for the PHQ-4 screening scale [19] for depression and anxiety were categorized as normal (0–2), mild (3–5), moderate (6–8), and severe (9–12). On each subscale, a score of 3 or greater was considered positive for screening purposes. As per recommended scoring procedures [23–26], PROMIS® raw scale scores for Anxiety, Depression, Anger and Peer Relationships were converted to standardized “T scores” (mean 50; SD 10) employing the Health Measures Scoring Service^SM^ [27] which utilizes norms for healthy 5 to 17-year children in the U.S. general population as the referent sample. For Anxiety, Depression and Anger, a higher score, for example T = 60, was representative of a + 1SD elevation in symptoms relative to the normative sample [23–25]. In the case of Peer Relationships, T = 60 reflected better social interactions by the same +1SD [26].

### Statistical analysis

Data analysis was performed using Minitab version 18 statistical software (Minitab LLC, State College, PA, USA). All data were described as medians and ranges (minimum, maximum); comparison between groups was performed by the Kruskal-Wallis test for continuous variables and subsequently adjusted for multiple comparisons using false discovery rates [28]. For DSD-specific and generic domains, the mean (SD) derived from previously validated reference data was used in the calculation of median SDS for each domain (Table 1). For DSD-specific domains, the median SDS of mothers and fathers within DSD and Endocrine groups were analysed to enable cross-parent comparisons. For all domains within both questionnaires, the median SDS of the DSD group was compared to the median SDS of the Endocrine group and the median SDS of each group (DSD and Endocrine) was also compared to SDS of zero. Mothers and fathers scores were combined for the PROMIS® measures as per scoring guidelines. A *p* value of less than 0.05 was considered statistically significant.

## Results

### Characteristics of DSD cases

For the Parent Self-Report (PSR) questionnaire, data were available for 55 parents (42 mothers and 13 fathers) of 54 children and for the Parent Proxy-Report (PPR) questionnaire, data were available for 25 parents (18 mothers and 7 fathers) of 25 children (Table 2). The median age of the 54 children for whom PSR questionnaires were completed was 1.8 years (range 0.03, 6.8 years) and of these, 44 (82%) were reared as boys. The median age of the 25 children for whom PPR questionnaires were completed was 4.5 years (2.0, 6.65) and of these, 16 (64%) were boys. Amongst the 54 DSD cases, 40 (74%) had 46, XY DSD with 28 (51%) having a diagnosis of a non-specific DSD (e.g. bilateral cryptorchidism or proximal hypospadias). Sex chromosome DSD and 46, XX DSD accounted for 7 (13%) cases each. Of the 54 cases, 16 (30%) had other conditions including cardiorespiratory disease, non-sex chromosome abnormalities or developmental delay.

**Table 2:** Sample characteristics of children with DSD and other Endocrine diagnoses

DSD sample	Parental Questionnaire	
	Self-Report^a^	Proxy-Report^b^
Index cases (*n)*	54	25
Parents (*n)*^c^	55	25
Mother	42	18
Father	13	7
Child gender (*n*, %)		
Male	44 (81.5%)	16 (64.0%)
Female	10 (18.5%)	9 (36.0%)
Child age, years (median, range)	1.8 (0.03, 6.8)	4.5 (2.0, 6.5)
DSD category and diagnosis		
Sex chromosome (*n*, %)	7 (13.0%)	6 (24.0%)
Turner syndrome	4 (7.4%)	4 (16.0%)
Other sex chromosome DSD (e.g. Klinefelter syndrome, 45X/46Y primary gonadal dysgenesis, XO/X isodicentric Y chromosome complement)	3 (5.5%)	2 (8.0%)
46, XX (*n*, %)	7 (13.0%)	6 (24.0%)
Disorder of androgen excess (congenital adrenal hyperplasia)	6 (11.1%)	5 (20.0%)
Other 46, XX DSD (e.g. vaginal atresia)	1 (1.9%)	1 (4.0%)
46, XY (*n*, %)	40 (74.0%)	13 (52.0%)
Disorder of gonadal (testicular) development (e.g. bilateral anorchia)	1 (1.9%)	1 (4.0%)
Non-specific disorder of undermasculinisation (e.g. bilateral cryptorchidism, isolated hypospadias)	28 (51.2%)	9 (36.0%)
Other 46, XY DSD (e.g. combination of hypospadias and bilateral cryptorchidism or micropenis)	11 (20.4%)	3 (12.0%)
Endocrine sample		
Index cases (*n)*	41	22
Parents (*n)*^d^	43	23
Mother	34	17
Father	9	6
Child gender (*n*, %)		
Male	23 (56.1%)	13 (59%)
Female	18 (43.9%)	9 (40.9%)
Child age, years (median, range)	2.0 (0.1, 6.8)	5.0 (2.3, 6.9)
Endocrine category (*n*, %)		
Disorders of short stature or growth hormone deficiency	14 (34%)	11 (50.0%)
Disorder of calcium and phosphate homeostasis	11 (26.9%)	4 (18.1%)
Disorder of thyroid gland	7 (17.1%)	4 (18.1%)
Disorder of adrenal gland	3 (7.3%)	2 (9.1%)
Disorder of pituitary gland	2 (4.9%)	0
Genetic disorders of glucose and insulin homeostasis	2 (4.9%)	0
Other (e.g. primary polydipsia, premature tooth exfoliation)	2 (4.9%)	1 (4.5%)

^a^Parent Self-Report (PSR) questionnaire completed by parents of children from birth to 2 years. ^b^Parent Proxy-Report (PPR) questionnaire completed by parents of children aged 2 to 6 years

^c^For one child, both parents completed PSR

^d^For two children, both parents completed PSR and for one child, both parents completed PPR

### Characteristics of endocrine cases

For the PSR questionnaire, data were available for 43 parents (34 mothers and 9 fathers) of 41 children and for the PPR questionnaire, data were available for 23 parents (17 mothers and 6 fathers) of 22 children (Table 2). The median age of the 41 children for whom parents completed PSR questionnaires was 2.0 years (0.1, 6.8) and of these 41 children, 23 (56%) were raised as boys. The median age of the 22 children for whom PPR questionnaires were available was 5.0 years (2.3, 6.9) and of these, 13 (59%) were boys. The most frequent endocrine diagnoses amongst the 41 children who had PSR questionnaires were conditions associated with short stature or growth hormone deficiency in 14 (34%) children and disorders of bone metabolism in 11 (27%) children. In the 22 children who had PPR questionnaires completed, these two diagnoses were present in 11 (50%) and 4 (18%) children, respectively. Other endocrine diagnoses in cases for whom PSR and PPR questionnaires were completed included thyroid disease (*n* = 7; *n* = 4), adrenal insufficiency (*n* = 3; *n* = 2), septo-optic dysplasia (*n* = 2), hyperinsulinism (*n* = 2), primary polydipsia (*n* = 1) and premature tooth exfoliation (*n* = 1); these diagnoses accounted for less than 7% of conditions amongst the endocrine cases. Of the 41 endocrine cases, 17 (41%), had other co-morbidity including cardiac, respiratory or neurological problems.

### Parent self-report scores - comparison to reference

**Fig. 2:**
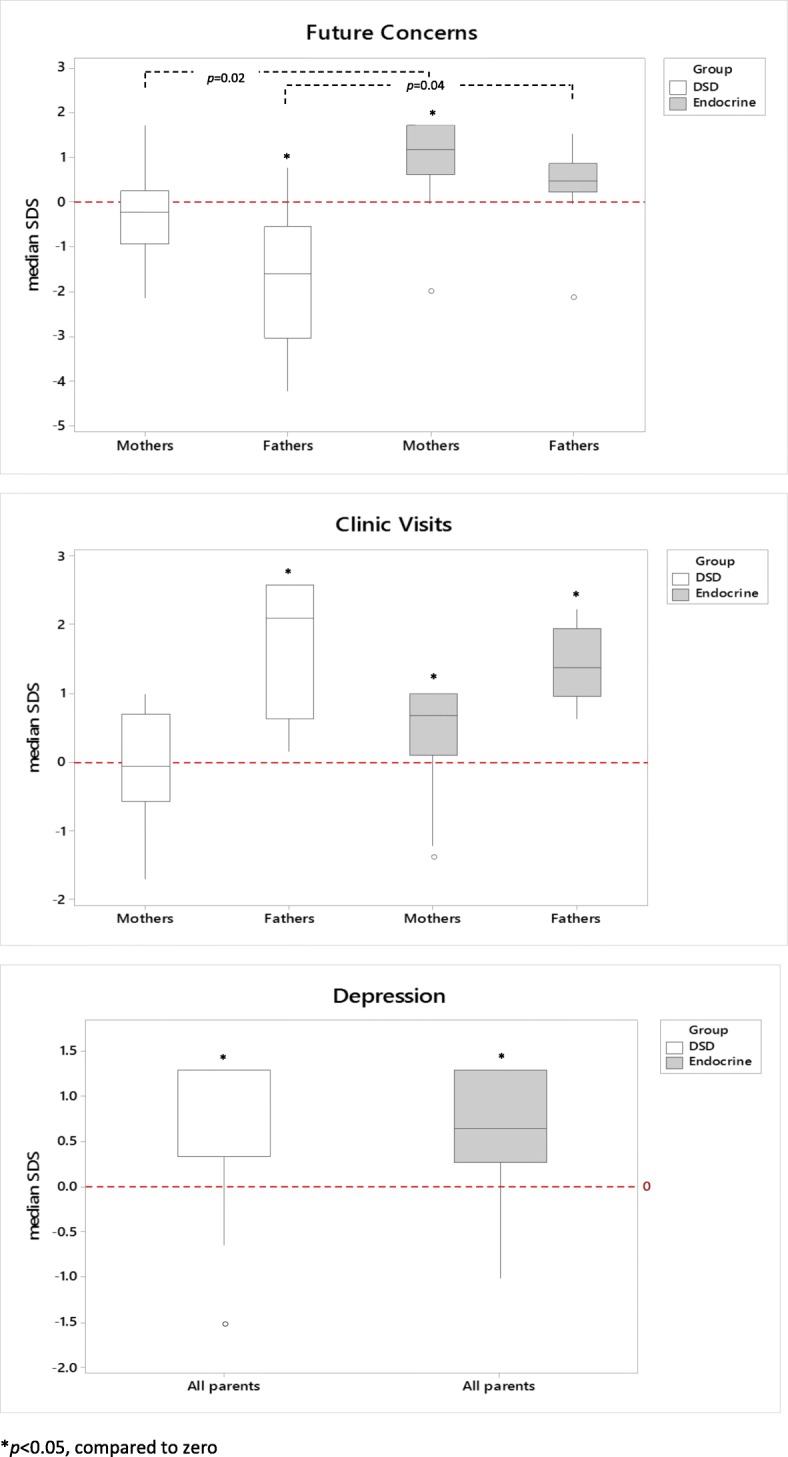
Boxplots for Future Concerns, Clinic Visits and Depression. DSD, Disorder of Sex Development; Endocrine, Children with other Endocrine conditions. *p < 0.05 represents a significant difference compared to zero

Fathers of children with DSD had lower median SDS for Future Concerns [SDS -1.60 (− 4.21, 1.00); *p* = 0.02], indicating greater apprehension, compared to reference data (Table 3, Fig. 2). However, these fathers had a higher median SDS for Medication [SDS 1.80 (1.01, 1.80); *p* = 0.04] and Clinic Visits [SDS 2.10 (0.16, 2.58); *p =* 0.02], indicating lesser degrees of stress relating to their child’s medication regimen and clinic visits. Mothers of children with other Endocrine conditions had a higher median SDS for Talking to Others [SDS 0.78 (− 0.83, 1.47); *p* = 0.02], Future Concerns [SDS 1.17 (− 2.00, 1.73); *p* = 0.02) and Clinic Visits [SDS 0.70 (− 1.37, 0.99); *p* = 0.02], indicative of less concerns in each of these domains. Fathers of children with other Endocrine conditions also had a higher SDS for Clinic Visits [SDS 1.37 (0.64, 2.60); *p* = 0.04], indicating less stress associated with clinic attendances, compared to reference data.

**Table 3:** Parent Self-Report questionnaire scores for children with DSD and children with other Endocrine conditions

Self-Report Domains	DSD Sample			Endocrine Sample			DSD vs Endocrine
	*n*^a^	Subscale score, median (range)	SDS^b^, median (range)	*n*^a^	Subscale score, median (range)	SDS^b^, median (range)	*p* value^c^
Communication and Information							
Mothers	42	75.00 (37.50, 100.00)	0.01 (−2.21, 1.49)	34	75.00 (25.00, 100.00)	0.01 (−2.95, 1.49)	NS
Fathers	13	75.00 (37.50, 87.50)	0.22 (−1.40, 0.76)	9	62.5 (0.00, 87.50)	−0.32 (−3.02, 0.76)	NS
Talking to Others							
Mothers	41	62.50 (35.00, 100.00)	−0.06 (− 1.18, 1.47)	34	83.13 (43.75, 100.00)	0.78* (− 0.83, 1.47)	NS
Fathers	12	81.25 (37.50, 100.00)	−0.25 (−2.86, 0.86)	9	90.00 (45.00, 100.00)	0.27 (−2.41, 0.86)	NS
Future Concerns							
Mothers	42	50.00 (0.00, 100.00)	−0.28 (−2.14, 1.73)	35	85.71 (3.57, 100.00)	1.17* (−2.00, 1.73)	0.02
Fathers	13	57.14 (21.43, 92.86)	−1.60* (−4.21, 1.00)	9	85.71 (50.00, 100.00)	0.48 (− 2.13, 1.52)	0.04
Medication							
Mothers	14	66.67 (0.00, 100.00)	−0.13 (−2.50, 1.05)	25	83.33 (0.00, 100.00)	0.31 (−2.50, 1.05)	NS
Fathers	6	100.00 (77.78, 100.00)	1.80* (1.01, 1.80)	3	100.00 (75.00, 100.00)	1.80 (0.92, 1.80)	NS
Clinic Visit							
Mothers	38	70.83 (56.25, 100.00)	−0.05 (−1.69, 1.00)	32	91.67 (33.33, 100.00)	0.70* (−1.37, 0.99)	NS
Fathers	11	87.50 (37.50, 100.00)	2.10* (0.16, 2.58)	8	68.75 (50.00, 91.67)	1.37* (0.64, 2.60)	NS
Surgery							
Mothers	11	12.50 (0.00, 91.67)	−1.02 (−1.51, 2.14)	4	31.30 (0.00, 83.30)	0.47 (−1.02, 1.80)	NS
Fathers	4	41.70 (25.0, 100.00)	−1.61 (−2.29, 0.77)	1	12.50 (12.50, 12.50)	−2.80 (− 2.80,-2.80)	NS
Stigma							
Mothers	42	1.60 (1.00. 2.67)	−0.25 (−1.21, 1.44)	35	1.40 (1.00, 3.20)	−0.57 (−1.21, 2.29)	NS
Fathers	13	1.40 (1.00, 2.10)	−0.36 (−1.27, 1.23)	9	1.60 (1.00, 2.11)	0.09 (−1.27, 1.25)	NS
PHQ-4							
Mothers	40	1.00 (0.00, 8.00)	0.54 (0.89, 1.96)	34	0.00 (0.00, 6.00)	0.89 (−1.25, 0.89)	NS
Fathers	12	0.00 (0.00, 6.00)	0.89 (0.89, 1.25)	9	0.00 (0.00, 4.00)	0.89 (−0.54, 0.89)	NS

^a^n’s vary by domain due to item responses and not all children have had surgery or take medication. ^b^*SDS* standard deviation score; subscale values are presented as SDS based on reference data. ^c^*p* values have been adjusted for multiple comparisons using false discovery rates; significance has been assigned at adjusted *p* < 0.05. *p < 0.05, compared to SDS of zero. All *p* values have been adjusted for multiple comparisons

### Parent self-report scores – comparison between DSD and endocrine groups

Mothers of children with DSD had lower median SDS for Future Concerns, indicating a greater level of concerns, than mothers of children with other Endocrine conditions [SDS -0.28 (− 2.14, 1.73) vs SDS 1.17 (− 2.00, 7.73); *p* = 0.02] (Table 3, Fig. 2). Similarly, fathers of children with DSD had a lower median SDS for Future Concerns compared with fathers of children with other Endocrine conditions [SDS -1.60 (− 4.21, 1.00) vs SDS 0.48 (− 2.13, 1.52); *p* = 0.04] indicating greater concerns in fathers of children with DSD. For both DSD and Endocrine groups, median PHQ-4 scores were not in the range associated with clinically significant symptomology and there was no significant difference in PHQ-4 scores between mothers and fathers of children with DSD compared with mothers and fathers of children with other Endocrine conditions (*p* > 0.05).

### Parent proxy-report scores- comparison to reference

Parents of children with DSD had higher median SDS for Depression [SDS 1.28 (− 1.52, 1.28); *p* = 0.03] indicative of greater depressive symptoms, compared to reference data (Table 4). Similarly, parents of children with other Endocrine conditions reported more symptoms of Depression [SDS 0.64 (− 1.01, 1.28); *p* = 0.03]. There was no significant difference in other Proxy-Report domains (including Anxiety, Anger and Peer Relations, Stigma, Clinic Visits and Medication) between parents of children with DSD or other Endocrine conditions, compared to reference data.

**Table 4:** Parent Proxy-Report questionnaire scores for children with DSD and children with other Endocrine conditions

Proxy-Report Domains	DSD Sample			Endocrine Sample			DSD vs Endocrine
	*n*^a^	Subscale score^b^, median (range)	SDS^c^, median (range)	*n*^a^	Subscale score^b^, median (range)	SDS, median (range)	*p* value^d^
Anxiety	23	43.60 (36.30, 68.60)	0.64 (−1.86, 1.37)	21	46.00 (36.30, 75.30)	0.40 (−2.53, 1.37)	NS
Depression	23	37.20 (37.20, 65.20)	1.28* (−1.52, 1.28)	22	43.60 (37.20, 60.10)	0.64* (−1.01, 1.28)	NS
Anger	23	43.40 (29.00, 62.70)	0.66 (−1.27, 2.10)	22	42.50 (29.00, 67.70)	0.75 (−1.77, 2.10)	NS
Peer Relations	23	50.60 (27.30, 60.80)	0.06 (−2.27, 1.08)	22	48.25 (19.10, 60.80)	−0.18 (−3.09, 1.08)	NS
Stigma							
Mothers	17	1.33 (1.00, 4.00)	−1.04 (−1.41, 1.89)	16	1.70 (1.00, 4.25)	−0.63 (−1.41, 2.17)	NS
Fathers	6	1.13 (1.00, 3.00)	−1.14 (− 1.30, 1.17)	6	1.75 (1.00, 2.50)	−0.37 (− 1.30, 0.56)	NS
Clinic Visit/ Medication							NS
Mothers	16	81.67 (3.33, 100.00)	0.68 (−2.52, 1.43)	17	93.30 (54.17, 100.00)	1.16 (−2.65, 1.43)	NS
Fathers	6	75.00 (40.00, 83.33)	−0.14 (−1.69, 0.23)	6	87.50 (66.67, 100.00)	0.42 (−0.51, 0.97)	NS

^a^n’s vary by domain due to item responses and not all children take medication. ^b^For anxiety, depression, anger and peer relations, the subscale score represents the PROMIS T-score. ^c^*SDS* standard deviation score, calculated using mean and SD from reference data. ^d^*p* values have been adjusted for multiple comparisons using false discovery rates; significance has been assigned at adjusted *p* < 0.05. *p < 0.05, compared to SDS of zero. All *p* values have been adjusted for multiple comparisons

### Parent proxy-report scores- comparison between DSD and endocrine groups

There was no significant difference in the PROMIS® scores for Anxiety, Depression, Anger and Peer Relationships between parents of children with DSD and parents of children with other Endocrine conditions (Table 4). In addition, there were no differences between parents of children with DSD compared with other Endocrine conditions with regards to DSD-specific domains including Stigma, Clinic Visits or Medications. Parents of children with DSD reported a median of 0 Missed School Days (0.0, 3.0) over the previous 6-month period compared to 0.5 days (0.0, 5.0) for children with other Endocrine conditions.

### Questionnaire acceptability

Of the 111 children whose parents were approached, the parents of 95 children with DSD or other Endocrine conditions completed the questionnaires (86%).In all cases, parents completed the questionnaires in less than 10 min and all parents reported that the questionnaire was acceptable for the time it took to complete the questionnaire, and questions were ‘easy to understand’ and ‘easy to follow’.

## Discussion

This is the first report of the use of brief patient-centered and parent-reported outcome tools, in parents and young children with DSD and a range of other Endocrine conditions that can be completed in less than 10 min as part of routine health care. It has demonstrated that parent self-report and parent proxy-report questionnaires are an acceptable practice in this context and can be routinely used in the outpatient setting to assess and monitor parent and patient needs. The rationale guiding the selection of the psychosocial domains to be included within our questionnaires was the same as that used for psychosocial assessments within the DSD-Translational Research Network [21] (i.e., that the measures deliver actionable information for the individual patient/family), with an additional requirement of reducing the time for questionnaire completion. The purpose of our assessment tools was to proactively monitor both patient and family psychosocial adaptation at a specific point in time enabling rapid quantification and insight into the experiences of children with DSD and other Endocrine conditions. Although the assessment approach adopted in selecting questionnaire items to characterize the adaptation of parents and young children with these chronic medical conditions was largely “non-categorical” [29], the finalized questionnaires included items that focussed on issues specific to, and shared by young patients born with DSD, and their families, that are not otherwise covered by generic health-related quality of life measures.

In the current report, questionnaires were introduced to patients following the clinic consultation; however, pre-clinic questionnaire completion and scoring would enable clinicians to review and act on results at the time of clinic visits and would increase their clinical utility. A quicker and simpler way of instantaneously displaying the results in the clinic setting could also increase the acceptability as well as the implementation of the tool [30]. The current report relates to evaluation of cross-sectional data from questionnaires collected from a large group of cases with a wide range of conditions within the umbrella of DSD. As a larger sample of cases with greater diagnostic homogeneity is collected with time, construct validity could be tested to determine the extent to which the questionnaires discrimate between groups that are known to differ on the items of interest. In the future, it will also be valuable to obtain longitudinal data to assess the responsiveness of the questionnaire by observing the temporal variation in outcomes in children with an assessment of factors (e.g. psychology services, timing of surgical interventions) that may influence a change in reported adaptation over time.

The questionnaires were designed for parents of children under the age of 7 years, thus, there is a need to develop similar tools for older children and adults. Furthermore, the questionnaires were developed in the English language, thus, lack of availability of the questionnaires in other languages may have provided a barrier for completion for some parents. The translation of questionnaires into other languages and their validation in different countries will need to be addressed in the future [31]. Nevertheless, the use of this quick and practical tool in the routine clinic setting raised some important and noteworthy observations.

Compared with parents of children with other Endocrine conditions, parents of children with DSD reported greater Future Concerns with regards to their child’s condition. These results highlight the need for ongoing parental support and effective communication between the multidisciplinary team and families [32] and the provision of early psychological input for parents of young children with DSD [12]. Despite these greater anxieties, fathers of children with DSD reported less stress with regards to attending for Clinic Visits and administering and managing Medication required for their child’s condition. It is commonly the case that mothers and fathers have different perceptions with regards to their child’s medical condition and perceive their child’s behaviour and emotions differently [33, 34]. It is unclear whether these differences are exaggerated in the context of DSD and our preliminary findings need to be confirmed in a larger sample size with a broad spectrum of DSD diagnoses. Approximately a quarter of cases of DSD are associated with multisystem co-morbidity [35]; similarly, in our current cohort, a third of children with DSD had other co-existing chronic conditions and the contribution of these co-morbidities to psychosocial well-being is not well established.

Parents of children with DSD and other Endocrine conditions reported more Depression compared to reference data; however, our results did not show any significant difference for any of the four PROMIS® domains (Anger, Anxiety, Depressive symptoms and Peer relationships) between parents of children with DSD and parents of children with other Endocrine conditions. Interestingly, for many domains including Healthcare Communication & Information and Stigma, scores were similar for parents of children with DSD and other Endocrine conditions, perhaps implying that parents of children with DSD have more positive experiences than anecdotal experience may suggest. Whilst there was no significant difference in the number of Missed School Days between DSD and Endocrine samples, this was an important domain for inclusion as previous studies have shown that children with chronic health conditions have greater school absenteeism and lower academic achievement than children who do not have chronic conditions [36, 37].

In summary, we have demonstrated that brief questionnaires are useful tools for collecting parent-reported psychosocial data for young children with DSD. The patterns of parental reporting observed can not only improve targeting of resources, but may also enable a greater understanding of the effect of the child’s condition on individual carers. A combination of generic and condition-specific scales may be more helpful than generic scales alone for identifying particular issues that need to be addressed in children with DSD and their families. Nevertheless, measures of cross-cutting domains (e.g., emotional state, behaviour problems, peer relations, etc) remain critically important because behaviour in these areas provide sine qua non evidence of adaptive or impaired psychological function. As such, scores on these latter measures can be used to compare adaptation and the influence of care strategies across specific DSD populations and against patients with other chronic paediatric conditions. Aggregating such data longitudinally and across multiple centres will create the opportunity for developing robust reference data which can be employed as clinical benchmarks [38] useful in guiding a process of continuous quality improvement in the care of these patients and their families.

## Conclusions

We present the first study reporting on the use of brief Parent Self-Report and Parent Proxy-Report questionnaires in a routine clinic setting for parents of young children with DSD. Preliminary results demonstrate that the screening approach followed is both feasible in the clinical context and acceptable to patients. Further, questionnaire scores showed variability across families and diagnostic categories; item responses suggested immediately actionable opportunities for counselling and clinical intervention. A broad consensus exists in the medical community regarding the influence of psychosocial aspects of DSD on patient and family outcomes. This report demonstrates that clinicians caring for these patients and their families can employ simple tools to identify psychosocial problems and tailor clinical recommendations accordingly.

## Supplementary information

**Additional file 1: Table S1.** Parent Self-Report questionnaire **Table S2.** Parent Proxy-Report questionnaire **Figure S1.** Parent Self-Report Questionnaire Information Sheet **Figure S2.** Parent Proxy-Report Questionnaire Information Sheet.

## Data Availability

The datasets used during the current study are available from the corresponding author on reasonable request.

## Abbreviations

DSD: Disorders/differences of sex development
PHQ-4: Patient health questionnaire-4
PPR: Parent proxy-report
PRO: Parent/patient-reported outcome
PROMIS®: Patient-reported outcomes measurement information system
PSR: Parent self-report
QOL-DSD-Parent: Quality of life dsd parent
QOL-DSD-Proxy: Quality of life dsd proxy
SDS: Standard deviation score
