# Supplementary material for: Parent-reported outcomes in young children with disorders/differences of sex development
Source: Int J Pediatr Endocrinol. 2020 Feb 14;2020:3. doi: 10.1186/s13633-020-0073-x (PMC7020572; doi:10.1186/s13633-020-0073-x)
Supplement: Supplementary file 1 — Additional file 1: Table S1. Parent Self-Report questionnaire Table S2. Parent Proxy-Report questionnaire Figure S1. Parent Self-Report Questionnaire Information Sheet Figure S2. Parent Proxy-Report Questionnaire Information Sheet. [file 13633_2020_73_MOESM1_ESM.docx]

**Supplementary Material**

1. Supplementary Table 1. Parent Self-Report questionnaire
2. Supplementary Table 2. Parent Proxy-Report questionnaire
3. Supplementary Figure 1. Parent Self-Report Questionnaire Information Sheet
4. Supplementary Figure 2. Parent Proxy-Report Questionnaire Information Sheet

**Supplementary Table 1. Parent Self-Report questionnaire**

| **Questionnaire Domain** | **Items** | **Scoring Options** |
| --- | --- | --- |
| **Healthcare Communication & Information** | I feel overwhelmed by the amount of information about my child’s condition  I hear confusing medical information about my child’s condition | Always true / Usually true / Sometimes / Seldom true / Never true |
| **Talking to Others** | I feel comfortable talking with my child about his/her condition^a^  I feel comfortable talking to close family members about my child’s condition  I am comfortable explaining my child’s needs (e.g. diaper changing, using the bathroom) to people other than family  I am not sure how much to tell others about my child’s condition  I worry about talking to others about my child’s condition because of how they might react | Always true / Usually true / Sometimes / Seldom true / Never true |
| **Experiences and Reactions** | I worry my child will look different from other teenagers or adults because of his/her condition  I worry my child won’t be/isn’t able to do things he/she wants to do because of their condition  I feel that I am odd or abnormal because of my child’s condition  There have been times when I have felt ashamed about having a child with this condition  I feel self-conscious about my child’s condition  People treat me the way they always have when they find out I have a child with this condition  I feel embarrassed about my child’s condition  People look down on me because I have a child with this condition  People say negative or unkind things about me behind my back because I have a child with this condition  I have been excluded from social gatherings because I have a child with this condition | Always true / Usually true / Sometimes / Seldom true / Never true |
| **Future Concerns** | I am concerned about how my child’s genitals will look  I am concerned about how my child’s genitals will function  I worry about my child dying due to the condition and/or its treatment  I worry that my child will have fertility issues (e.g. will not be able to have a biological child)  I worry that my child will have social problems, like being teased about his/her condition  I worry about my child’s future relationships (e.g. dating, marriage)  I worry that my child will not be comfortable with his/her gender as an adult | Always true / Usually true / Sometimes / Seldom true / Never true |
| **Medications** | How much stress do you experience …  Remembering to give your child his/her medications related to the condition (e.g. hormones such as growth hormone, steroids, thyroxine, testosterone)?  Making sure your child receives his/her medications for the condition when he/she is away from you (e.g. at school or daycare)?^a^  Giving your child his/her medication?  Managing the side effects of your child’s condition? | A great deal / Moderate / Some / None / N/A No medication  Option to select ‘no medication taken’ |
| **Visit to the Endocrine Clinic** | How long ago was your child’s most recent endocrine clinic visit?  How much stress did you experience …  Talking with your child before the visit?^a^  Not knowing what to expect at the visit?  Managing your child’s behaviour during the visit? | Today-2 weeks ago / 2 weeks- 3 months ago / 3- 6 months ago / 6- 12 months ago / 1-2 years ago / Over 2 years ago  A great deal / Moderate / Some / A little / None / N/A |
| **Surgery**^b^ | How long ago was your child’s most recent surgery related to the condition?  How much stress did you experience …  Talking with your child before the surgery?^a^  During the surgery?  After the surgery (e.g. dealing with your child’s needs for extra care, wondering about the outcome)? | Today-2 weeks ago / 2 weeks- 3 months ago / 3- 6 months ago / 6- 12 months ago / 1-2 years ago / Over 2 years ago  A great deal / Moderate / Some / A little / None / N/A |
| **PHQ-4** | Over the past 2 weeks, how often have you been bothered by the following problems:  Little interest or pleasure in doing things  Feeling down, depressed or hopeless  Feeling nervous, anxious or on edge  Not being able to stop or control worrying | Not at all / Several days / More than half the days / Nearly every day |

^a^Option to omit question by selecting option ‘too young’

^b^Option to select ‘no surgery’

**Supplementary Table 2. Parent Proxy-Report questionnaire**

| **Questionnaire Domain** | **Items** | **Scoring Options** |
| --- | --- | --- |
| **Anxiety** | My child felt nervous  My child felt worried  My child felt like something awful might happen  My child worried when he/she was at home | Never / Almost Never / Sometimes / Often / Almost Always |
| **Depressive Symptoms** | My child felt everything in his/her life went wrong  My child felt lonely  My child felt sad  It was hard for my child to have fun | Never / Almost Never / Sometimes / Often / Almost Always |
| **Anger** | My child felt mad  My child was so angry he/she felt like yelling at somebody  My child was so angry he/she felt like throwing something  My child felt upset  When my child got mad, he/she stayed mad | Never / Almost Never / Sometimes / Often / Almost Always |
| **Peer Relationships** | My child felt accepted by other kids his/her age  My child was able to count on his/her friends  My child and his/her friends helped each other out  Other kids wanted to be my child’s friend | Never / Almost Never / Sometimes / Often / Almost Always |
| **Experiences and Reactions** | People who know that my child has the condition treat him/her differently  It really doesn’t matter what I say to people about my child’s condition, they usually have their minds made up  In many people’s minds, having this condition attaches a stigma or label to my child  Because of the condition, my child will have problems in finding a boyfriend or girlfriend (husband or wife) | Always true / Usually true / Sometimes / Seldom true / Never true |
| **Visit to the Endocrine Clinic** | How long ago was the endocrine clinic visit or procedure?  What did the endocrine clinic visit or procedure involve? (write in)  How much stress did your child experience …  Before the endocrine clinic visit or procedure (e.g. on way to the appointment, in the waiting room)?  Having endocrine clinic visits e.g. physical exams?  Having doctors examine the private parts of my child’s body?  Having medical procedures (e.g. blood tests)?  After the visit/procedure? | Today-2 weeks ago / 2 weeks- 3 months ago / 3- 6 months ago / 6- 12 months ago / 1-2 years ago / Over 2 years ago  A great deal / Moderate / Some / None / N/A No exam |
| **Medications** | How much stress did your child experience  Taking medication for the condition? | A great deal / Moderate / Some / None / N/A No medication |
| **Missed School Days** | Over the past six months, excluding school holidays, how many days of school has your child missed because of the condition he/she attends the clinic for? | Number of days |

**Supplementary Figure 1. Parent Self-Report Questionnaire Information Sheet**

**Paediatric Endocrine Clinic Wellbeing Questionnaire**

(for children aged 0- 7 years)

Understanding the effects of your child’s medical condition on everyday life gives us information about how to support your family directly. For this reason, we have developed a questionnaire specifically for parents of children with conditions that requires them to attend the **Endocrine Clinic**.

This questionnaire is based on questions that often arise in the clinic but either due to time restraints or other reasons were not covered fully in the clinic.

In this questionnaire, we are interested in finding out about **your** feelings and experiences relating to your child’s endocrine condition.

We refer to “your child’s condition” throughout this questionnaire, we are referring to your child’s **endocrine condition** for which they are attending the clinic, and not to any other medical conditions that he or she may have. *The questionnaire should not take more than 10 minutes of your time.*

We will store this information in your child’s case records with all the other details of the clinic visit, and, as always, the information that you provide through this questionnaire is **confidential.**

This questionnaire is a new addition to our service and please feel free to advise us on how we can improve our service or this questionnaire in the section at the end of the questionnaire.

**Instructions:** Please answer all the questions by checking one box for each question. There are no right or wrong answers! If you are not sure how to answer, choose the response that seems closest to your situation.

Please mark **not applicable (“N/A”)** only if the question is not relevant for one of two reasons: (a) because your child is either too young for the question to apply, or (b) if you or your child never had the experience asked about in the question (e.g., mark “N/A” for surgery questions if your child has never had surgery).

***Please enclose the questionnaire in the attached envelope and hand it to the front desk at the endocrine clinic***

| *Insert patient sticker* | Date of clinic:  Consultant: |
| --- | --- |
| **Person completing questionnaire**  Relationship to patient:  Mother □  Father □  Other: ______________  ^please describe^ | ^check one below^  Completed before seeing doctor □  Completed after seeing doctor □  Date of completing questionnaire:  _ / / _  ^Day Month Year^ |

**Supplementary Figure 2. Parent Proxy-Report Questionnaire Information Sheet**

**Paediatric Endocrine Clinic Wellbeing Questionnaire**

(for children aged 2-6 years)

Understanding the effects of your child’s medical condition on everyday life gives us information about how to support your family directly. For this reason, we have developed a questionnaire specifically for parents of children with conditions that requires them to attend the **Endocrine Clinic**.

This questionnaire is based on questions that often arise in the clinic but either due to time restraints or other reasons were not covered fully in the clinic.

In this questionnaire, we are interested in finding out about **your child’s** feelings and experiences relating to their endocrine condition.

We refer to “your child’s condition” throughout this questionnaire, we are referring to your child’s **endocrine condition** for which they are attending the clinic, and not to any other medical conditions that he or she may have. *The questionnaire should not take more than 10 minutes of your time.*

We will store this information in your child’s case records with all the other details of the clinic visit, and, as always, the information that you provide through this questionnaire is **confidential.**

This questionnaire is a new addition to our service and please feel free to advise us on how we can improve our service or this questionnaire in the section at the end of the questionnaire.

**Instructions:** Please answer all the questions by checking one box for each question. There are no right or wrong answers! If you are not sure how to answer, choose the response that seems closest to your situation.

Please mark **not applicable (“N/A”)** only if the question is not relevant for one of two reasons: (a) because your child is either too young for the question to apply, or (b) if you or your child never had the experience asked about in the question (e.g., mark “N/A” for surgery questions if your child has never had surgery).

***Please enclose the questionnaire in the attached envelope and hand it to the front desk at the endocrine clinic***

| *Insert patient sticker* | Date of clinic:  Consultant: |
| --- | --- |
| **Person completing questionnaire**  Relationship to patient:  Mother □  Father □  Other: ______________  ^please describe^ | ^check one below^  Completed before seeing doctor □  Completed after seeing doctor □  Date of completing questionnaire:  _ / / _  ^Day Month Year^ |
